# Three-dimensional scoring of zebrafish behavior unveils biological phenomena hidden by two-dimensional analyses

**DOI:** 10.1038/s41598-017-01990-z

**Published:** 2017-05-16

**Authors:** Simone Macrì, Daniele Neri, Tommaso Ruberto, Violet Mwaffo, Sachit Butail, Maurizio Porfiri

**Affiliations:** 1 Centre for Behavioural Sciences and Mental Health, Istituto Superiore di Sanità, Viale Regina Elena 299, 00161 Roma, Italy; 2 Department of Mechanical and Aerospace Engineering, New York University, Tandon School of Engineering, 6 MetroTech Center, Brooklyn, NY 11201 USA; 3 Department of Mechanical Engineering, Northern Illinois University, DeKalb, IL 60115 USA

## Abstract

The study of zebrafish behavior represents a cornerstone upon which basic researchers promise to advance knowledge in life sciences. Although zebrafish swim in a three-dimensional (3D) space, their behavior in the lab is almost exclusively scored in two dimensions, whereby zebrafish are recorded using a single camera providing 2D videos. Whether this dimensional reduction preserves the reliability of data has not been addressed. Here we show that, compared to a 3D observation, 2D data are flawed by over-reporting and under-reporting of locomotory differences. Specifically, we first reconstructed 3D trajectories through the integration of synchronous information derived from two cameras, and then compared them with the original 2D views in classical experimental paradigms assessing shoaling tendency, fear, anxiety, and general locomotion. Our results suggest that traditional behavioral scoring of individual zebrafish performed in 2D may undermine data integrity, thereby requiring a general reconsideration of scoring zebrafish behavior to incorporate a 3D approach. We then demonstrate that, compared to 2D, a 3D approach requires a reduced number of subjects to achieve the same degree of validity. We anticipate these findings to largely benefit animal welfare by reducing the number of experimental subjects, without affecting statistical power.

## Introduction

The surge in the use of zebrafish in preclinical research^1–4^ - motivated by their neurobiological proximity to human beings, sequenced genome, rapid reproduction rate, and elevated stocking density - has been paralleled by a rapid increase in behavioral test paradigms^1, 2, 4, 5^. These paradigms rest upon a detailed analysis of individual behavior in isolation or in interaction with live and/or artificial stimuli^6–9^. Animal behavioral studies entail conducting experiments, video recording of the experimental sessions, and detailed scoring of the videos. Most of these studies rely on 2D data, whereby the analysis is performed on videos recorded using a single camera^10, 11^. Yet, in their ethological niche, zebrafish exhibit complex 3D swimming patterns^12^, that are dependent on their anisotropic appraisal of the environment^13^ and mediated by different portions of the peripheral nervous system^14, 15^.

To quantify the loss of information due to 2D analysis compared to 3D, we analyzed a large set of data, collated from our previous studies^16, 17^ and new experiments conducted ad hoc. Comparing locomotory patterns, and assuming 3D information to be the ground-truth, we scored a false negative finding if a statistically significant difference was found between two given conditions in 3D but not in 2D. Correspondingly, if we observed a statistically significant difference in 2D but not in 3D, we scored a false positive finding. The database consisted of time-resolved trajectories of 90 adult zebrafish tested in canonical binary choice preference experiments with the following stimuli: live conspecific, live predator, 3D printed model of a conspecific, computer animated image of a predator, and 3D printed model of a predator. These tests have been frequently used by independent authors to investigate fundamental biological domains, such as anxiety, memory, fear, and general locomotion^6–9, 18^. The comparative analysis was afforded by a 3D scoring method that we originally developed to quantify zebrafish locomotory patterns^16^. This method combines the information derived from two cameras, positioned orthogonal to one-another to provide a top and a front view of zebrafish swimming^16^. Following the synchronization of the two videos, the 3D approach integrates the position information from the top and front views^16^.

We first experimentally substantiated the prediction that 2D (top and front) views underestimate swimming activity compared to 3D measurements. Then, most importantly, we evaluated whether 2D views yield false (positive or negative) results by investigating between-group differences in swimming activity. Since 3D scoring accounts for all locomotion-related parameters (full path length, exact position in the tank, and accurate swimming speed), we considered these data to constitute a ground truth against which testing the reliability of experimental data collected in 2D. Specifically, we evaluated between-group differences in all experimental conditions in 3D, and then used this information to estimate the number of false negative and positive findings yielded by 2D top and front views.

## Results and Discussion

**Figure 1:**
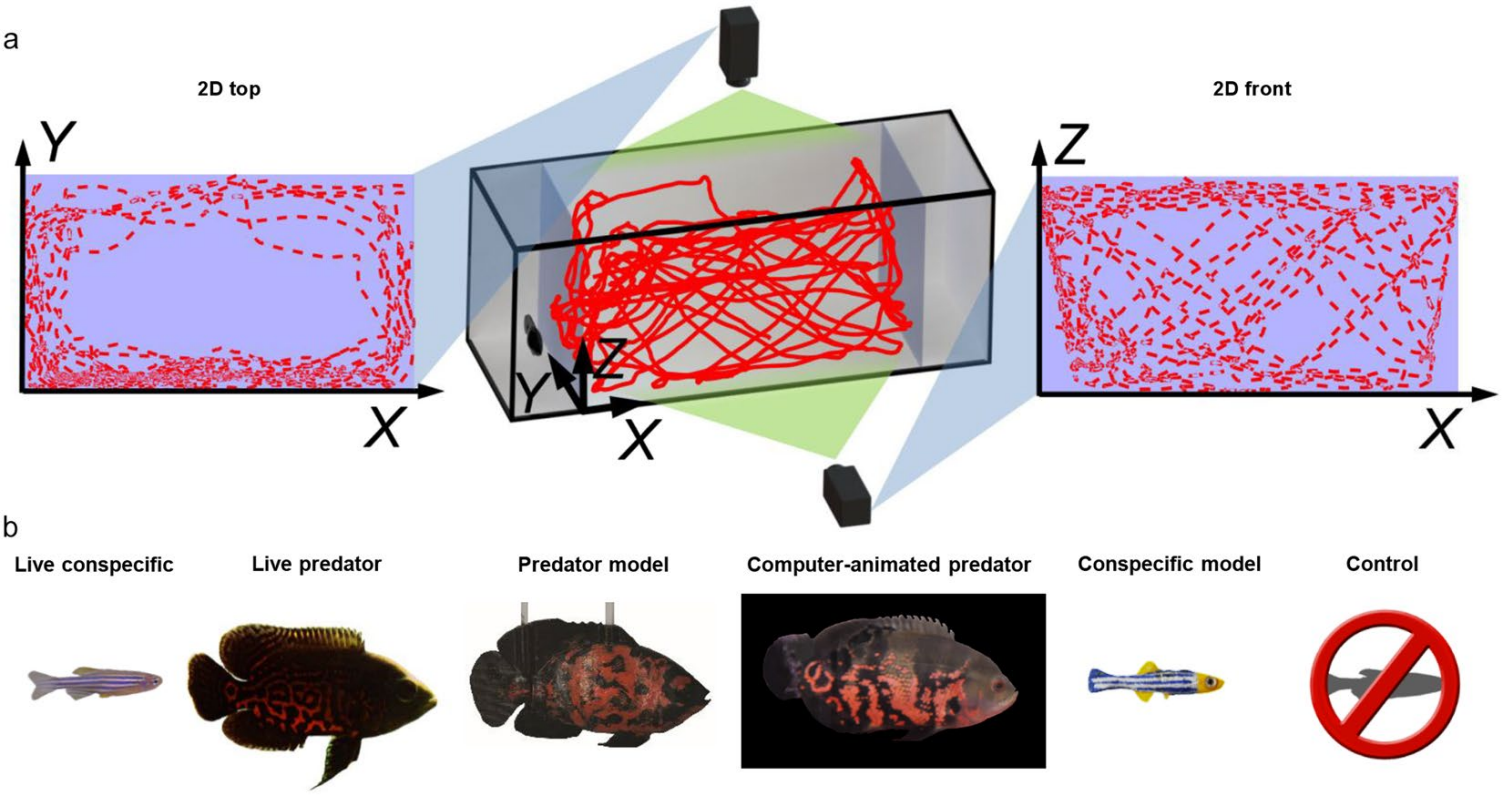
Schematics of the setup to acquire 2D and 3D trajectories in response to the experimental stimuli. (**a**) Representative image of the 3D reconstructed trajectory obtained through the synchronization of 2D data from top (left) and front (right) views. (**b**) Images of the stimuli utilized in the six experimental conditions comparing individual preference for an empty compartment with preference for a compartment containing one of the following stimuli: live conspecific (LC); live predator (LP), red tiger oscar fish; predator model (PM), 3D-printed model of red tiger oscar fish anchored in the stimulus compartment and beating its tail through an external motor; computer-animated predator (CAP), animated image of red tiger oscar fish projected on a computer screen; conspecific model (CM), 3D-printed model of zebrafish actuated by a robotic platform to move along realistic 3D trajectories and oscillate its body; empty compartment, control (CTRL).

To demonstrate that 3D absolute values are different from 2D, we quantified zebrafish average speed, average peak speed, average angular speed, average peak angular speed, and wall following in the six experimental conditions (see Fig. 1; here and henceforth, the word “average” refers to an arithmetic mean computed by summing data from a single trial performed by an individual and dividing by the number of frames of the trial). In five of these conditions, we also evaluated average distance from the stimulus (live conspecific, live predator, their respective robotic models, and computer-animated predator), thereby gathering data on spatial preference.

### 2D views may lead to inaccurate measurement of swimming activity

**Figure 2:**
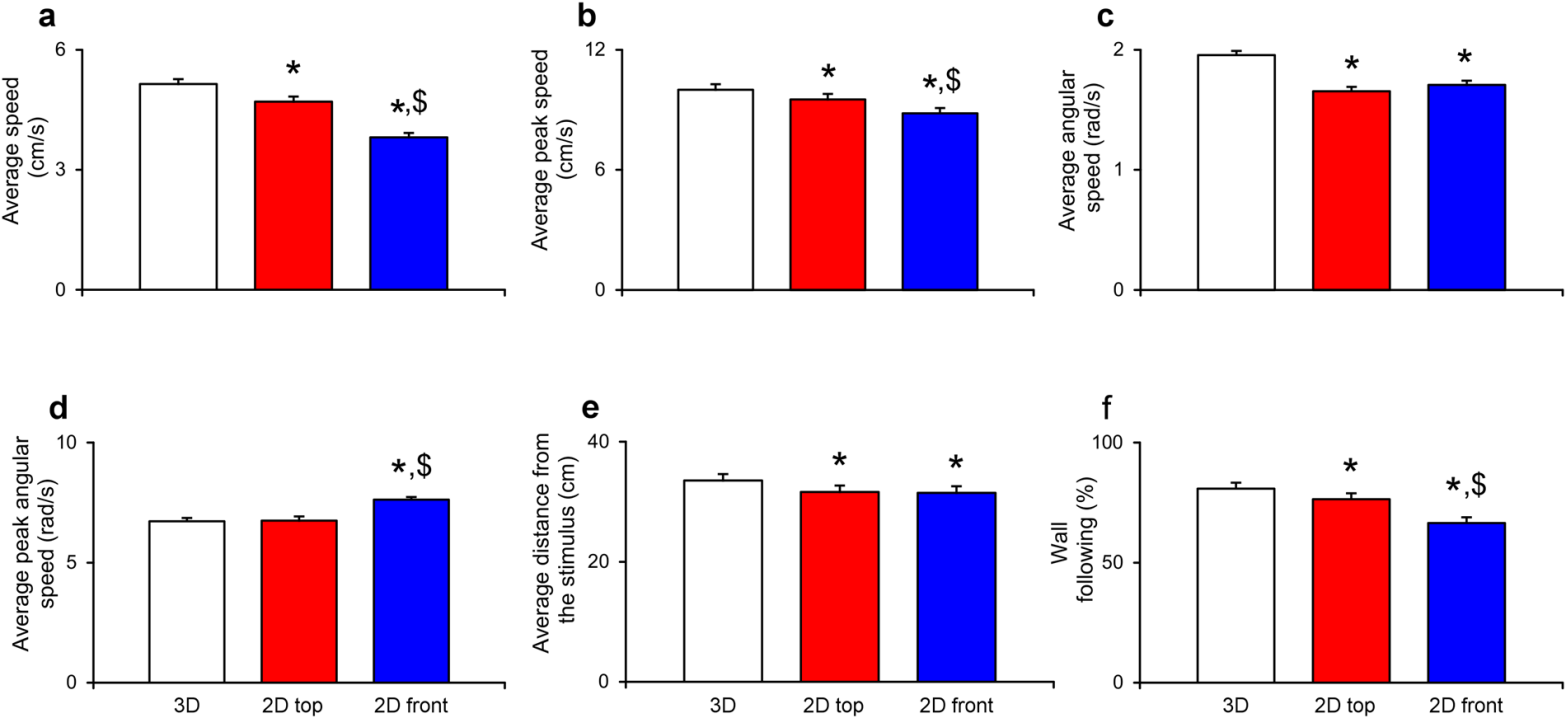
Comparison between 3D reconstructed trajectories and 2D top and front views. Group values of average speed, average peak speed, average angular speed, average peak angular speed, wall following, and average distance from the stimulus depending on the scoring method: “3D” represents the 3D reconstructed data, “2D top” represents 2D data acquired from the top camera, and “2D front” represents 2D data acquired from the front camera. Data were analyzed through repeated measures ANOVA for split-plot designs with condition as between-subject factor and scoring method as within-subject factor. Post-hoc comparisons were performed using Tukey’s HSD with a significance level of 0.05: * Identifies significant differences in comparisons with 3D measurements and $ identifies significant differences with 2D top views. Data are expressed as mean + standard errors. (**a**) Average speed values varied with the scoring method (n = 90; F_2,168_ = 247.434, p < 0.001): 3D view yielded higher values compared to 2D top and front views; furthermore, 2D top view values were higher than 2D front view values. (**b**) Average peak speed values varied with the scoring method (n = 90; F_2,168_ = 26.118, p < 0.001): 3D view yielded higher values compared to 2D top and front views; furthermore, 2D top view values were higher than 2D front view values. (**c**) Average angular speed values varied with the scoring method (n = 90; F_2,168_ = 51.905, p < 0.001): 3D view yielded higher values compared to 2D top and front views. (**d**) Average peak angular speed values varied with the scoring method (n = 90; F_2,168_ = 44.160, p < 0.001): 2D front view yielded higher values compared to 3D and 2D top views. (**e**) Values of average distance from the stimulus varied with the scoring method (n = 80; F_2,150_ = 127.409, p < 0.001): 3D view yielded higher values compared to 2D top and front views. (**f**) Time spent following the walls of the tank varied with the scoring method (n = 90; F_2,168_ = 71.531, p < 0.001): 3D view yielded higher values compared to 2D top and front views; furthermore, 2D top view values were higher than 2D front view values.

Predictably, we observed that both 2D views underestimated 3D tracking (see Fig. 2) with respect to average speed, average peak speed, average angular speed, and wall following. These discrepancies should be attributed to the truly 3D nature of the swimming tank in which zebrafish are allowed to exhibit their rich behavioral repertoire^12^, composed of maneuvers along the three axes. Downscaling such maneuvers on arbitrary 2D views leads to a remarkable, perspective-dependent, reduction in the measured length of each locomotion bout contributing to the average speed, average peak speed, average angular speed, average distance from the stimulus, and wall following.

The overestimation of the average peak angular speed in 2D front view compared to 2D top view and 3D should be attributed to an underestimation of the radius of curvature of fish trajectories during U-turns, which mainly take place in the horizontal plane. From the front view, U-turns – with a true radius of curvature comparable with the fish body length – will appear as sudden changes of swimming direction of nearly 180 degrees, thereby leading to extreme bursts in the angular speed. Not only is swimming activity underestimated in 2D compared to 3D, but 2D views also yield inconsistent results. Specifically, the reduced locomotion characterizing the front view compared to the top view could be related to the differential exhibition of diving movements along the water column with respect to in-plane swimming^19^.

The underestimation of fish swimming activity co-occurred with a consistent overestimation of the spatial preference for the stimulus, whereby the average distance from the stimulus was consistently underestimated in 2D measurements. While this is partly related to underestimating general locomotion, an arbitrary correction factor is unlikely to make the results more realistic. Substantiating this, the differences in average speed between 2D views do not translate into variations in the average distance from the stimulus.

Preventing the exhibition of unwanted behaviors through shallow or narrow water tanks^20^ may not constitute the countermeasure of choice as it hampers the natural behavioral repertoire of zebrafish, thereby potentially masking biologically relevant phenomena. For example, anxiety-related behaviors encompass both horizontal (escape) and vertical (diving) movements^12^: providing narrow or shallow experimental tanks would respectively thwart the former or the latter. Thus, the dimensional reduction afforded by 2D behavioral scoring may constitute a methodological concern, should the selective prevention of given locomotory patterns (for example vertical or horizontal movements) mask the effects of experimental treatments.

### 2D views may yield false results

**Figure 3:**
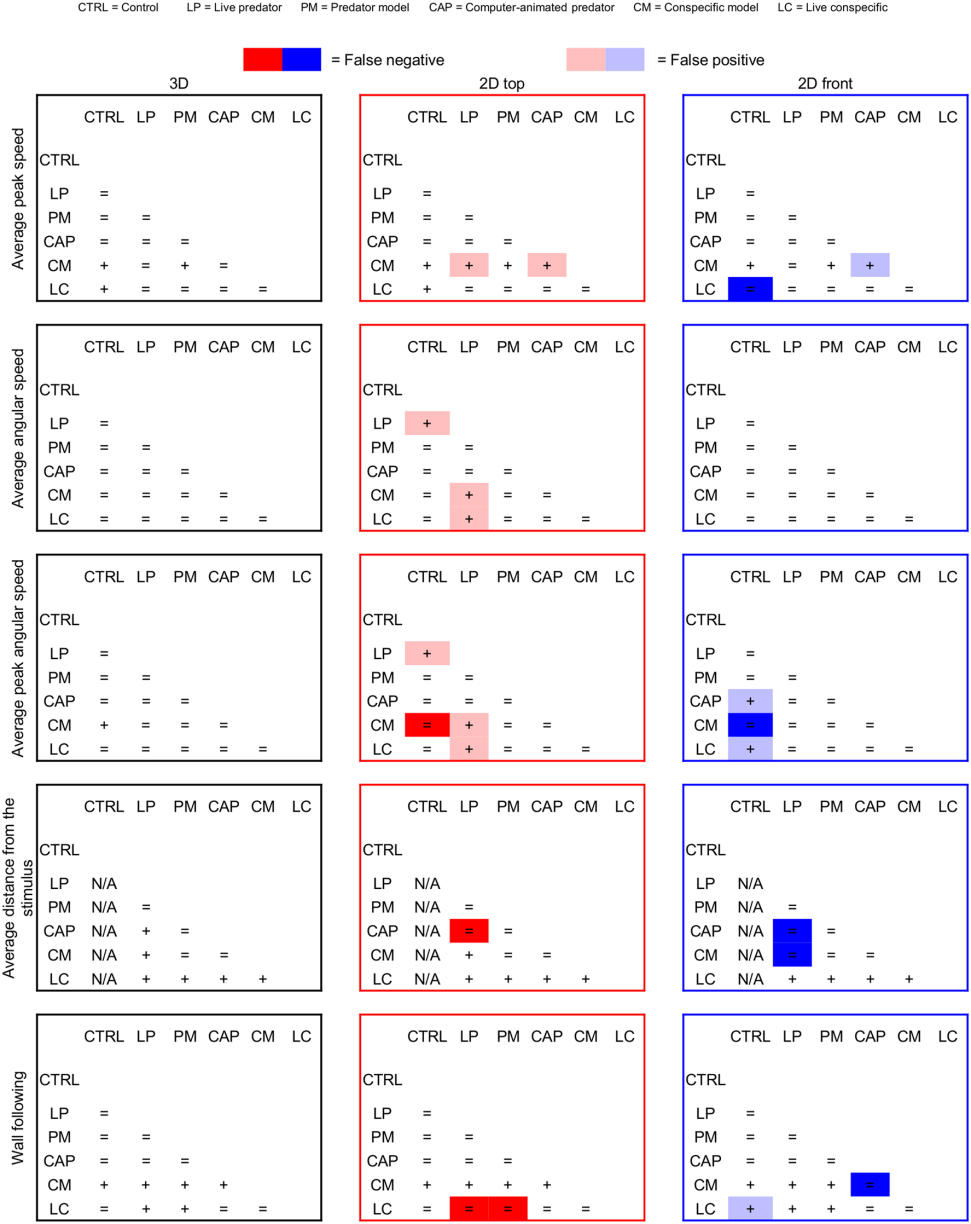
Synopsis of the condition x scoring method interactions demonstrating that differences between conditions vary depending on the scoring method. Repeated measures ANOVA comparisons for average peak speed, average angular speed, average peak angular speed, average distance from the stimulus, and wall following for each of the six experimental conditions, with data collected using 3D reconstruction (black panels), 2D top (red panels), and 2D front (blue panels) views. The statistical model was a repeated measures ANOVA for split-plot designs with condition (CTRL, LP, PM, CAP, CM, LC) as between-subject factor and scoring method (3D, 2D top, and 2D front) as within-subject factor. We observed significant interactions between condition and scoring method in all the following parameters: average peak speed (n = 90; F_10,168_ = 2.131, p < 0.05); average angular speed (n = 90; F_10,168_ = 4.209, p < 0.001); average peak angular speed (n = 90; F_10,168_ = 4.260, p < 0.001); average distance from the stimulus (n = 80; F_8,150_ = 11.026, p < 0.001); wall following (n = 90; F_10,168_ = 6.305, p < 0.001) respectively. + symbols indicate significant differences in post-hoc comparisons (p < 0.05 in Tukey’s HSD) between the corresponding conditions in each contingency table. Dark red and blue boxes highlight false negative results, and light red and blue boxes highlight false positive results using 3D data as ground truth reference.

To demonstrate the possibility that group differences varied depending on the use of 3D or 2D tracking, we studied the interaction between the scoring method and experimental conditions (see Fig. 3). Beside the identification of significant main effects of condition and scoring method, the split-plot statistical model permitted demonstration of significant condition x scoring method interactions in all the parameters considered, with the exclusion of average speed (n = 90; F_10,168_ = 2.492, p = 0.0733). Therefore, the effects of experimental conditions varied depending on the methodology adopted to score individual behavior.

**Figure 4:**
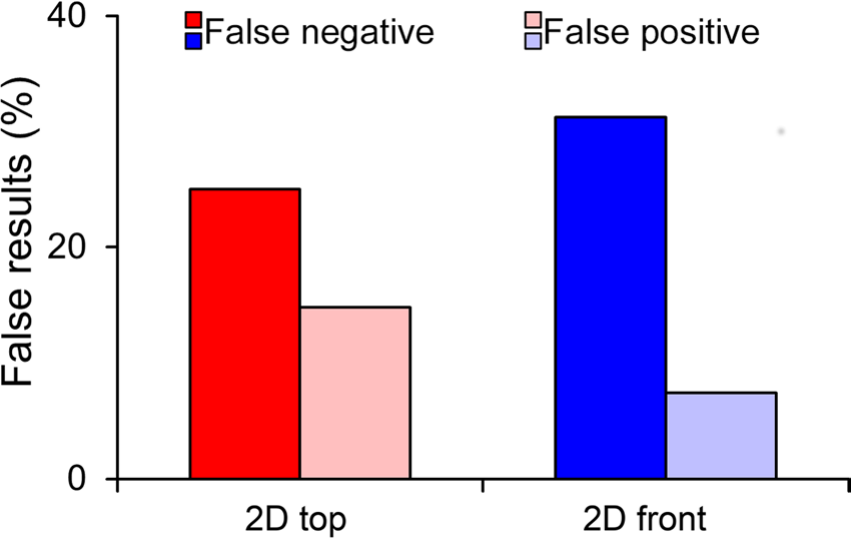
Effect of 2D downscaling on false negative and positive results. Percentage of false (negative and positive) results comparing 2D views and 3D tracking in terms of average peak speed, average angular speed, average peak angular speed, average distance from the stimulus, and wall following. The percentage of false results was computed by dividing the number of false negative (resp. positive) results with the corresponding number of valid comparisons in the 3D data. In particular, if the number of significant differences in 3D were D_s_ and non-significant D_n_, then the percentage of false negatives for a given view was computed as (number of false negatives)/D_s_, and correspondingly the percentage of false positives was computed as (number of false positives)/D_n_. Data from Fig. 3 were pooled to quantify the proportion of false results obtained from 2D top (red bar) and front (blue bar) views. False negative results are identified with dark red and blue, and false positive results are identified with light red and blue.

Assuming the 3D approach to constitute the closest approximation to the general population (ground truth data), we calculated the number of false positive and false negative findings in 2D top and front views. In light of the presence of significant condition x scoring method interactions in average peak speed, average angular speed, average peak angular speed, average distance from the stimulus, and wall following, we performed Tukey’s honest significant difference (HSD) post-hoc tests to pairwise quantify which conditions were significantly different from the others (this post-hoc test accounts for the multiple testing by adjusting the level of significance according to the exact number of relevant contrasts); we finally compared the pairwise matrix of 3D data with those obtained using 2D front and top views. This comparison revealed that 2D views yielded an elevated number of false negative and positive results with the former outweighing the latter (see Fig. 4). Evidence for the possibility of false results using 2D scoring methods is also offered in the study of Stewart *et al*.^18^, through the comparison of nicotine-treated versus control zebrafish tested in the novel tank test. In line with our statistically-based analysis, the authors suggest the possibility of an interaction effect between the scoring method and nicotine treatment.

The condition x scoring method interactions cannot be attributed to differential datasets, whereby the 2D trajectories used in the analyses were the same that, combined, constituted the 3D trajectory. This result supports our hypothesis that 2D downscaling attenuates group differences through an unwarranted distortion of biologically-relevant locomotory patterns. For example, the increase in the average peak speed due to the presence of a live predator was not revealed by 2D tracking from the front view. This suggests that the increase in the average peak speed, detected by the top view and confirmed by 3D tracking, may relate to more frequent horizontal movements, masked from the front camera. We thus propose that the dimensional reduction is responsible for the drop in statistical power, as it fails to account for the behavioral complexity inherent to zebrafish physiology.

### A 3D approach may help reducing the number of experimental subjects

The elevated number of false negative results (25% for top view and 31.25% for front view) indicates that 2D data are underpowered compared to 3D (see Fig. 4). Thus, to attain analogous statistical results, 2D approaches would likely require an increased number of observations/experimental subjects. To calculate the differential power of 3D and 2D approaches we studied the sensitivity of 3D tracking to the sample size. Specifically, we determined the total number of false negatives and false positives that would result from a reduction in the sample size of the experiment.

We systematically reduced the sample size from n = 90 to 30 and focused on the parameters for which we identified a significant condition x scoring method interaction: average peak speed, average angular speed, average peak angular speed, average distance from the stimulus, and wall following. To ensure an unbiased sample size reduction, we randomly selected fish to be discarded and iterated the process 100 times. We assembled all the pairwise comparisons in a binary vector and computed its Hamming distance^21^ from the reference vector representing all the pairwise comparisons of the original experimental sample (this distance corresponds to the total number of false results).

**Figure 5:**
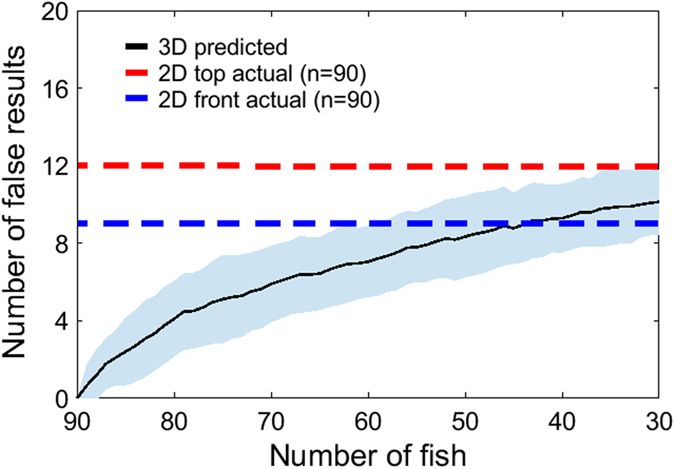
Effect of reduced sample size on false negative and positive results. Quantification of the loss in statistical power in 3D tracking as a function of the reduction in sample size. The analysis was conducted by progressively and randomly (100 repetitions) reducing the population size available to 3D tracking from its full size (average, solid line; standard deviation, shaded area) of n = 90 to 30. First, the 70-dimensional binary vector aggregating pairwise differences from the first column in Fig. 3 was assembled. Then, the sample size was reduced and an equivalent vector was generated. The number of false results due to the random and progressive reduction was quantified through the Hamming distance between the two vectors. Our results show that the Hamming distance increases monotonically, with a new false result being generated on average every sixth removed fish (computed from the start and endpoint of the black line). Dashed lines represent the number of false results obtained in 2D front (blue) and top (red) tracking with full sample size (n = 90). These numbers (as reported in Fig. 3) are equal to 12 for 2D top and 9 to 2D front, respectively, whereby 2D top view yields 4 false negative and 8 false positive results and 2D front view 5 false negative and 4 false positive results. Our findings show that the statistical power of 2D tracking on a dataset comprising 90 individuals is comparable to that possessed by 3D tracking over a population comprised of half the subjects.

Our computations indicate that the Hamming distance increases monotonically with the reduction in sample size, causing on average a new false result every six removed fish. Thus, half of the population analyzed through 3D tracking would beget approximately the same number of false results of 90 individuals scored using 2D tracking from any of the two views (see Fig. 5). Increased statistical power of 3D tracking may result in a remarkable reduction in the number of zebrafish required in preclinical studies, thereby fulfilling one of the key requests of ethical animal research^22^.

## Conclusion

Three-dimensional scoring of behavior comes at the cost of using multiple cameras, adjusting the experimental setup to accommodate angled views and increasing the computational load to handle and fuse the large amount of data^16, 19, 23–26^. 2D approaches have played a pivotal role in elucidating the neurobehavioral underpinnings of collective behavior, across a range of powerful experimental paradigms. For example, a number of studies based on 2D approaches have demonstrated that collective behavior may consistently vary as a function of fear- and anxiety-eliciting stimuli^5^ or pharmacological manipulations^27^. Besides reiterating the fundamental advancements provided by 2D scoring, we believe that future studies are needed to evaluate the extent to which 3D scoring may complement the analysis of collective behavior. Specifically, it is necessary to test whether the limitations of 2D observed in individually-tested experimental subjects extend to experiments in which groups of subjects are tested together. In fact, the present data pertain to single individuals tested in preference experiments, thereby requiring future studies on shoaling and schooling^28–30^. While 3D tracking systems may be used in the study of small shoals of zebrafish, future work should seek to test whether it may benefit experimental validity and animal welfare. 

Ultimately, rather than merely suggesting that 3D scoring shall replace traditional studies, we propose a synergistic integration of 2D and 3D approaches to favor technical and theoretical scientific advancements. While a 2D approach should suffice to accurately score several zebrafish locomotory patterns, it is important to anticipate potentially spurious data that may come with it and clarify when a 3D approach should be employed.

## Materials and Methods

We analyzed data from six different experiments where fish were filmed from two orthogonal views. These included a new experiment with a live conspecific (LC) as stimulus, data from the experiments in ref. 16 with live predator (LP), computer-animated predator (CAP), and 3D printed predator model (PM) as stimuli, a control condition (CTRL) with no stimuli, and data from a single condition in ref. 17, with the fish model moving in 3D (CM) as stimulus.

### Ethics statement

Experiments with live conspecifics (LC) and experiments from ref. 17 were performed in accordance with the relevant guidelines and regulations, and were approved by the University Animal Welfare Committee (UAWC) of New York University under protocol number 13–1424 (Robotics based experimental paradigms to study social behavior, reward pathways, and their development adjustments in fish).

Experiments from ref. 16 were performed in accordance with the relevant guidelines and regulations, and were approved by the University Animal Welfare Committee (UAWC) of New York University under protocol number 13–1424, and the Animal Welfare Oversight Committee of the Polytechnic Institute of New York University (now New York University Tandon School of Engineering which was independent from New York University until 2013 with respect to animal care) under protocol number AWOC-2013-103.

### Animals and housing

Data from all three studies (current, refs 16 and 17) comprise a total of 90 experimentally naïve adult zebrafish (*Danio rerio*) of wild-type variety and with an approximate average body length of 3 cm. All fish were purchased from the same vendor (LiveAquaria.com, Rhinelander, WI, USA). Fish were housed in 37.8 L (10 gallon) tanks, and were given a period of 12 to 15 days to acclimate to the housing tanks prior to the start of experiments. Acidity and temperature were maintained at 7.2 pH and 26 °C. The two red-tiger oscar fish (*Astronotus ocellatus*), used as live predators in ref. 16, measured approximately 8 cm in body length. These were housed in a separate 115 L (31 gallons) holding tank with analogous water conditions. Lights in the animal facility were automatically turned on at 9:00 AM and turned off at 9:00 PM, resulting in a 12 h light/12 h dark photoperiod^31^. While zebrafish were fed with commercial flake food (Hagen Corp. Nutrafin max, Mansfield, MA, USA) between 6:00 pm and 7:00 pm every day, the oscar fish were fed with commercial shrimp (Zoo Med Laboratories, Inc., San Luis Obispo, CA, USA) and commercial flake food (Hagen Corp.Nutrafin max, Mansfield, MA, USA) at approximately 7:00 pm every day. Fish across all experiments were used only once. Experiment-specific apparatuses and procedures for each of the studies are detailed below.

### Current experiment: zebrafish versus live conspecific

#### Apparatus

The apparatus consisted of a 74 cm long, 30 cm wide and 30 cm deep tank. The tank was filled with tap water to a depth of 27 cm, and treated with Stress Coat conditioner (API Mars Fishcare, Chalfont, PA, USA). The temperature of the water was maintained at 26 °C. The internal walls and floor of the tank were covered with white contact paper, with the exception of one long side, allowing the recording of the experiments from the front. Two square Plexiglas panels (30 cm long and 1 cm thick) were used to divide the tank in three compartments: one central compartment (54 cm) and two lateral compartments (10 cm). The tank was surrounded by black curtains in order to avoid any interaction with the surrounding environment. Two 25 W fluorescent tubes (All-Glass Aquarium, UK) were mounted along the long sides of the tank to illuminate it from 13 cm above the water level.

Two Flea3 USB cameras (Point Grey Research Inc., Richmond, British Columbia, Canada) were used to record videos triggered at the same time, one from the front and the other from the top. This allowed the 3D reconstruction of the movement of the fish. Each camera was positioned 50 cm away from the tank and pointed towards the center of the tank. A laser light was pointed toward a corner in the empty partition of the tank for few seconds within the first ten seconds of recording of each session to verify the synchronization of the videos recorded by the two cameras.

#### Experimental procedure

The trials took place in May 2016. For this experiment, 20 zebrafish were selected and randomly allocated to constitute the experimental group (ten focal subjects) or the stimulus group (ten live stimuli). Fish were gently hand-netted from their housing tanks to the experimental tank. The live stimulus was placed in one of the lateral partitions, and then the focal fish was placed in the central section. The position of the stimuli in the lateral partitions was randomized between trials and balanced throughout the photoperiod.

The cameras started recording within a second after the release of the focal fish in the central partition. The recordings included 10 min of habituation time, which were discarded for analysis, and 10 min of experimental observation, for a total of 20 min.

### Experiment in Ref. 16

#### Apparatus

Similar to the live conspecific study, the apparatus consisted of a rectangular tank of the same dimensions, subdivided into three compartments using Plexiglas panels. White contact paper was applied to the rear wall and to the bottom of the tank, while leaving another long side clear for filming. LCD display screens were positioned along the tank width, oriented toward the center of the tank. Illumination was provided by two 25 W fluorescent tubes (All-Glass Aquarium, UK) mounted along the longitudinal sides of the tank, 16 cm above the water surface. The apparatus was isolated using black curtains to avoid any disturbance.

Two web cameras (Logitech Webcam Pro 9000, Logitech, Newark, CA, USA), mounted 65 cm away from the tank, were used to record videos from top and front view. Recording was triggered for both cameras simultaneously using a Linux shell script at 25 fps. A laser light was pointed intermittently outside the tank, while keeping it in the camera views, in order to synchronize the top and front videos.

#### Robotic predator model

The biologically-inspired robotic predator model was designed to mimic the red-tiger oscar fish in its morphology, color pattern and tail beat frequency. Briefly, the predator model measured a maximum of 9.5 cm length and 4.7 cm height (dorsal fin to pectoral fin), and was designed in SolidWorks (Dassault Systèmes SolidWorks Corp., Waltham, MA, USA) and 3D printed in acrylonitrile butadiene styrene (ABS) thermoplastic using a Dimension SST 3D printer (Stratasys Ltd., Eden Prairie, MN, USA). The color pattern was created with waterproof spray paints (Krylon, Krylon Products Group, Cleveland, OH, USA).

The predator model was anchored in a fixed position in the stimulus compartment to a 3 mm-thick Plexiglas plate placed over the tank (along the vertical axis of the stimulus compartment). Two 22 cm-long transparent Plexiglas rods (frontal and rear) were used to suspend the robotic fish 5 cm from the bottom of the tank. While the frontal rod was held fixed, the rear one was actuated by an external servomotor (HS 82-MG, Hitec RCD USA, Inc., Poway, CA, USA), controlled by a microcontroller (Arduino Uno, Arduino, Italy), to give the appearance of tail movement. Following visual measurement of the tail-beat of the live oscar fish, the servomotor parameters were set at 1 Hz and 21°, respectively for its angular frequency and its maximum angle of rotation.

#### Computer-animated stimulus

The computer-animated stimulus consisted of an image of a red tiger oscar (measuring 9 cm in length, approximately the size of the live predator and the predator model) on a dark background, displayed on a computer screen. Two 17-inches monitors (Dell E177FPc LCD Monitor, Round Rock, TX, USA) were utilized to provide the computer-animated stimuli. The red tiger oscar picture was moved at a speed of 0.7 cm/s along a sinusoidal trajectory that had an amplitude of 1 cm and a frequency of 0.4 Hz. The computer animation looped throughout the entire trial, taking care that the Oscar appeared and disappeared gradually and from different locations on the screen.

#### Experimental procedure

A total of 70 zebrafish were utilized for the experiments. Specifically, 20 fish were used for each of the live predator (LP), computer-animated predator (CAP), and predator model (PM) condition, and 10 fish were used for the CTRL condition. The experiments were performed between August 2013 and March 2014, and consisted of five trials performed in the morning and afternoon, for a total of 10 trials per day. For each condition, a focal fish was hand netted from its housing tank and placed in the central section of the tank for 20 min, comprising of 10 min of habituation and 10 min of experimental time. Black plastic panels were used to cover the lateral partitions and manually removed at the end of the habituation period. The two computer screens displayed a blank black screen in the conditions LP, PM, and CTRL. In each experimental condition that involved presenting a stimulus, its position was counterbalanced between the two lateral sides of the tank across trials.

### Experiment in Ref. 17

#### Apparatus

In this experiment, we used the same three-partitioned tank utilized in the current study and in ref. 16 and the water level was set at 15 cm. Two web cameras (Logitech Webcam Pro 9000, Logitech, Newark, CA, USA) were placed 55 cm away from the tank in each direction to record from top and front views. Illumination was provided by two 25 W fluorescent tubes (All-Glass Aquarium, UK) mounted 30 cm above the water surface, along the long sides of the tank.

#### Conspecific fish model and robotic platform

The conspecific fish model (CM) resembled zebrafish morphology (including dorsal, ventral, and caudal fins), and was fabricated using ABS thermoplastic in a Dimension Elite 3D printer (Stratasys Ltd., Eden Prairie, MN, USA). The model was painted with blue, silver, and yellow non-toxic colors (Krylon, Krylon Products Group, Cleveland, OH, USA) to mimic live zebrafish striped pattern. Glass eyes (Van Dyke Supply Co., Granite Quarry, NC, USA) were used to increase the conspecific model attractiveness toward the live fish^32^.

The conspecific model was attached to an acrylic rod, which was actuated in three dimensions through a four-degree-of-freedom (three independent translations along the three axes and one oscillation about the vertical axis) robotic platform, that actuated the fish model along a pre-programmed trajectory, reconstructed from a live zebrafish swimming pattern^17^. The robotic arm was driven by two servomotors (Futaba Corporation of America, Schaumburg, IL, USA), a DC motor (Robotzone, LLC, Winfield, KS, USA), and a stepper motor (Adafruit, New York City, NY, USA), for translation motion, and another servomotor (Hitec RCD USA, Inc., Poway, CA, USA) for oscillation. The platform was capable of implementing complex swimming behavior such as freezing, thigmotaxis, erratic movement, and diving. The average speed of the model replica was 1.69 cm/s, and it was positioned on average at 7.56 cm under the water surface.

#### Experimental procedure

The experiments took place between August and September 2015. A total of 10 naïve zebrafish were tested in this condition, five in the morning and five in the afternoon. Briefly, the focal fish was released in the central section, with robotic platform and CM in one compartment and an empty compartment on the other side. Post-release, the cameras were triggered simultaneously using a Linux shell script to record at 30 frames per second for 20 min, 10 min each for habituation and observation. The presentation of the fish model replica actuated with the platform was counterbalanced between the two lateral sides during the experimental sessions.

### Video post-processing

Recorded videos were converted into frames, and post-processed using the 3D multi-target tracking algorithm presented in ref. 16, implemented in MATLAB R2014b (Mathworks, Natick, MA, USA), for all conditions except CM. For CM, each of the front and top views were tracked separately and 2D data were merged to create 3D estimates. Therein, a simple moving average was applied on the position time series using a window size of 18 frames.

### Data analysis

To evaluate the differences between 3D and 2D motion, all the parameters were computed using the data from the 3D reconstructed data and from the 2D data (top and front). Fish swimming activity was scored in terms of average speed, average peak speed, average angular speed, average peak angular speed, average distance from the stimulus, and wall following. These parameters were computed as follows: average speed was computed by averaging the magnitude of the first derivative of the position time series; average peak speed was estimated as the average of the speed values greater than the 90^th^ percentile in the overall speed distribution; average angular speed in 2D was computed from a central difference approximation of the curvature of fish trajectories^33^, by summing the absolute values of clockwise or counterclockwise rotations; a similar procedure was implemented to estimate the average angular speed in 3D, where instantaneous curvature was computed in the plane specified by three successive positions; average peak angular speed was calculated as the average of the absolute values of the angular speed greater than the 90^th^ percentile of the angular speed distribution; average distance from the stimulus was computed as the mean distance from the stimulus position over all frames; and wall following was computed as the time spent within one body length (3 cm) of distance from the walls (lateral and bottom) of the tank.

Two-way ANOVA was used to compare the five of the six parameters (average speed has been excluded from this analysis) in each of the six examined conditions, with the condition (LC, LP, PM, CTRL, CM, and CAP) as between-subjects factor and scoring method (3D, 2D top, 2D front) as within-subject factor. In case of significant condition x scoring method, a Tukey’s HSD post-hoc test was utilized for pairwise comparisons. This analysis led to 15 pairwise comparisons for each single parameter and each data type, with the exception of average distance from the stimulus where only 10 pairwise comparisons were performed, for a total of 140 pairwise comparisons. Each single pairwise comparison was assigned a “+” sign in case the parameter was found to significantly differ between conditions, and an “=” sign when they were not found to vary between conditions. These comparisons were utilized to identify false positive results (light red for top and light blue for front) and false negative results (red for top and blue for front) (see Fig. 3). Considering 3D data to be ground truth, a false positive was counted for a given parameter if there was no significant difference in that parameter between conditions, but a significant difference was found with 2D data; conversely, a false negative was counted if a significant difference was found with 3D data but no such difference was found in 2D data.

Pairwise post-hoc comparisons between conditions for a parameter where significance was found were further utilized to compute the Hamming distance^21^ between data from either 2D top or 2D front and 3D, for average peak speed, average angular speed, average peak angular speed, average distance from the stimulus, and wall following. Specifically, a binary vector was created using the pairwise comparisons between all conditions for each parameter, with a 1 denoting significant difference and 0 otherwise. This resulted in a vector length of 70 binary values (15 pairwise comparisons x 5 parameters, excluding the 5 pairwise comparisons for the distance from the stimulus in control condition) for each view. The Hamming distance between the 3D and each of the two 2D views was then computed as the total number of pairwise comparisons that were different between the two datasets. Note that this is the same as the total number of false positives and false negatives reported for a particular dataset (2D top or front).

We further conducted a statistical analysis on 3D reconstructed data to identify the effects of the sample size on the Hamming distance for average peak speed, average angular speed, average peak angular speed, average distance from the stimulus, and wall following. In particular, we randomly reduced the 3D population size from its full size to a progressively smaller population of 3D tracked individuals (smallest population size n = 30). Each successive decrease of the population was iterated 100 times. We then computed Tukey’s HSD post-hoc test to differentiate between conditions, followed by the Hamming distance between the original 3D dataset and the reduced sample-size 3D dataset. All the analyses were conducted with a significance level set at p < 0.05.

Datasets and codes used in the analyses are stored at the authors’ home institution and will be provided on request.
